# Human Atrial Fibroblast Adaptation to Heterogeneities in Substrate Stiffness

**DOI:** 10.3389/fphys.2019.01526

**Published:** 2020-01-10

**Authors:** Ramona Emig, Callum M. Zgierski-Johnston, Friedhelm Beyersdorf, Bartosz Rylski, Ursula Ravens, Wilfried Weber, Peter Kohl, Maximilian Hörner, Rémi Peyronnet

**Affiliations:** ^1^Institute for Experimental Cardiovascular Medicine, University Heart Center Freiburg - Bad Krozingen, Medical Center-University of Freiburg, Freiburg, Germany; ^2^Faculty of Medicine, University of Freiburg, Freiburg, Germany; ^3^Faculty of Biology, University of Freiburg, Freiburg, Germany; ^4^Department of Cardiovascular Surgery, University Heart Center Freiburg, University of Freiburg, Freiburg, Germany; ^5^Signalling Research Centres BIOSS and CIBSS, University of Freiburg, Freiburg, Germany

**Keywords:** fibrosis, mechanosensing, hydrogel, light-tunable, nanoindentation

## Abstract

Fibrosis is associated with aging and many cardiac pathologies. It is characterized both by myofibroblast differentiation and by excessive accumulation of extracellular matrix proteins. Fibrosis-related tissue remodeling results in significant changes in tissue structure and function, including passive mechanical properties. This research area has gained significant momentum with the recent development of new tools and approaches to better characterize and understand the ability of cells to sense and respond to their biophysical environment. We use a novel hydrogel, termed CyPhyGel, to provide an advanced *in vitro* model of remodeling-related changes in tissue stiffness. Based on light-controlled dimerization of a Cyanobacterial Phytochrome, it enables contactless and reversible tuning of hydrogel mechanical properties with high spatial and temporal resolution. Human primary atrial fibroblasts were cultured on CyPhyGels. After 4 days of culturing on stiff (~4.6 kPa) or soft (~2.7 kPa) CyPhyGels, we analyzed fibroblast cell area and stiffness. Cells grown on the softer substrate were smaller and softer, compared to cells grown on the stiffer substrate. This difference was absent when both soft and stiff growth substrates were combined in a single CyPhyGel, with the resulting cell areas being similar to those on homogeneously stiff gels and cell stiffnesses being similar to those on homogeneously soft substrates. Using CyPhyGels to mimic tissue stiffness heterogeneities *in vitro*, our results confirm the ability of cardiac fibroblasts to adapt to their mechanical environment, and suggest the presence of a paracrine mechanism that tunes fibroblast structural and functional properties associated with mechanically induced phenotype conversion toward myofibroblasts. In the context of regionally increased tissue stiffness, such as upon scarring or in diffuse fibrosis, such a mechanism could help to prevent abrupt changes in cell properties at the border zone between normal and diseased tissue. The light-tunable mechanical properties of CyPhyGels and their suitability for studying human primary cardiac cells make them an attractive model system for cardiac mechanobiology research. Further investigations will explore the interactions between biophysical and soluble factors in the response of cardiac fibroblasts to spatially and temporally heterogeneous mechanical cues.

## Introduction

Cardiac tissue damage due to causes such as aging, mechanical overload, or injury is associated with the development of fibrosis. Fibrosis is characterized both by myofibroblast differentiation and excessive accumulation of extracellular matrix (ECM) proteins (Herum et al., 2017). This remodeling is driven mainly by fibroblasts (Manabe et al., 2002; Travers et al., 2016), changes in tissue structure, and mechanics with consequences ranging from impaired cardiac output to increased arrhythmia vulnerability (Nguyen and Qu, 2014). Ultimately, presence and extent of fibrosis are a leading risk factor for sudden cardiac death (Disertori et al., 2016). Thus far, there are no effective therapeutic approaches toward reversing cardiac fibrosis mainly due to insufficient knowledge about the underlying basic mechanisms.

Possible therapeutic approaches must consider changes in tissue mechanics, which act both as a cause and a consequence of remodeling. Fibroblast sensing of their mechanical environment has been shown to play a role in fibrotic remodeling (Hinz, 2013; van Putten et al., 2016). In recent years, several *in vitro* models have been developed to study fibroblast mechanosensing. A major class of these models utilizes synthetic substrates that include hydrogels and silicones. These allow investigators to prescribe the mechanical environment of cultured cells (Rosales and Kristi, 2016; Li et al., 2018). Alternatively, naturally occurring substrates like decellularized tissue (Ott et al., 2008) or living cardiac tissue slices (Perbellini et al., 2018) have been used to provide cells with near-physiological growth substrates, but these are more complex and less reproducible models. Substrates that more closely mimic *in vivo* conditions are essential as, while the overall stiffness of fibrotic tissue is raised when ECM production overcomes degradation (Levental et al., 2010), the stiffness distribution is highly heterogeneous. Areas with more or less ECM constitute different mechanical microdomains. In diffuse fibrosis, for example, in atrial fibrillation, islands of stiff ECM are distributed within softer tissue (Tanaka et al., 2007). Given that the mechanical effects on single cells are dominated by their microenvironment, *in vitro* models should replicate these stiffness heterogeneities, ideally in a controllable manner.

A model allowing the introduction of spatial gradients in the mechanical properties has been used to show that the stiffness-guided migration of fibroblasts, a process called durotaxis, depends on the type of matrix proteins (Hartman et al., 2017). Further, hydrogels with micropatterned stiff and soft areas were used to show adaptation of cell spreading over a wide range of stiffnesses (Sunyer et al., 2012) and to study the role of matrix organization for mesenchymal stem cell differentiation (Yang et al., 2016). However, these models do not allow one to dynamically and reversibly modify passive mechanical properties of the growth substrate in time or space. A novel hydrogel system (which we termed here CyPhyGel), based on the Cyanobacterial Phytochrome Cph1, may address this limitation by allowing light-controlled, reversible changes in hydrogel stiffness (Hörner et al., 2019b). To do so, cell-compatible red light is used to switch Cph1 between its monomeric (740 nm) or dimeric (660 nm) forms, thereby reducing or increasing the number of crosslinks in the growth substrate in a contact-free manner, respectively. The stiffness of CyPhyGels can be changed between 1.5 and 5.5 kPa (Effective Young’s modulus). Changes in stiffness occur within seconds of illumination, are gradable, stable in the absence of light, and reversible (Hörner et al., 2019b). In this study, we use CyPhyGels to investigate human cardiac fibroblast adaptation to heterogeneities in the stiffness of their mechanical environment beyond durotaxis.

## Materials and Methods

### Production of Cph1-Based Hydrogels (CyPhyGels)

CyPhyGels were produced as described previously (Hörner et al., 2019b) with the following modifications. In short, the photoreceptor Cph1* [Cph1-Y263F (amino acids 1–514) fused to a tandem arginine-glycine-aspartic acid motif, a hexahistidine tag and C-terminal cysteine] was recombinantly produced by high-cell-density fermentation in *E. coli* BL21 STAR (DE3) and purified *via* immobilized metal ion affinity chromatography (Hörner et al., 2019a). Purified Cph1* was concentrated to ~200 mg/mL by ultrafiltration (PES membrane, 10 kDa molecular weight cutoff). The elution buffer from purification was exchanged with reaction buffer (phosphate buffered saline [PBS], containing, in mM 137 NaCl, 2.7 KCl, 10 Na_2_HPO_4_, 1.8 KH_2_PO_4_, 2 mM ethylenediaminetetraacetic acid, pH 8) using a desalting column (5 kDa molecular weight cut-off). After concentrating the protein as before to ~100 mg/mL, it was reduced with tris(2-carboxyethyl)phosphine (TCEP) at a molar Cph1*:TCEP ratio of 1:0.7 for 1 h at room temperature. Without removing the reducing agent, the protein was covalently coupled to 8-arm polyethylene-glycol vinyl-sulfone (PEG-VS, 40 kDa, NOF Europe GmbH, Germany) in reaction buffer supplemented with 100 mM triethanolamine at a final concentration of 70 mg/mL Cph1* and a molar VS:Cph1* ratio of 2:1.

Immediately after addition of PEG-VS, 30 μL of the reaction mix were spread on 22 mm × 22 mm square glass coverslips, resulting in CyPhyGels with a thickness between 50 and 90 μm (data not shown). The reaction mixes were incubated in a humidified atmosphere to allow gelation at room temperature for at least 16 h under continuous illumination with 660 nm (1 mW/cm^2^). Afterward, CyPhyGels on coverslips were transferred into PBS (pH 7.4) and stored in the dark at room temperature. Their mechanical properties after different illumination protocols (see “Stiffness Tuning of CyPhyGels”) were tested by nanoindentation (see “Nanoindentation of CyPhyGels and Fibroblasts”). CyPhyGels were used no earlier than 1 week after preparation, as their stiffness increased over several days following gelation (Supplementary Figure S1).

### Stiffness Tuning of CyPhyGels

Light emitting diodes (LED) with peak wavelengths of 660 nm to stiffen CyPhyGels, and 740 nm to soften CyPhyGels (LZ4-40R208 and LZ4-40R308, respectively; LED Engin, USA) were coupled to bandpass filters restricting specimen illumination to ± 6.5 nm around the target wavelength (FF01–660/13 and FF01–740/13, respectively; Semrock, USA). CyPhyGels were illuminated at 1 mW/cm^2^ for a minimum of 5 min.

**Figure 1:**
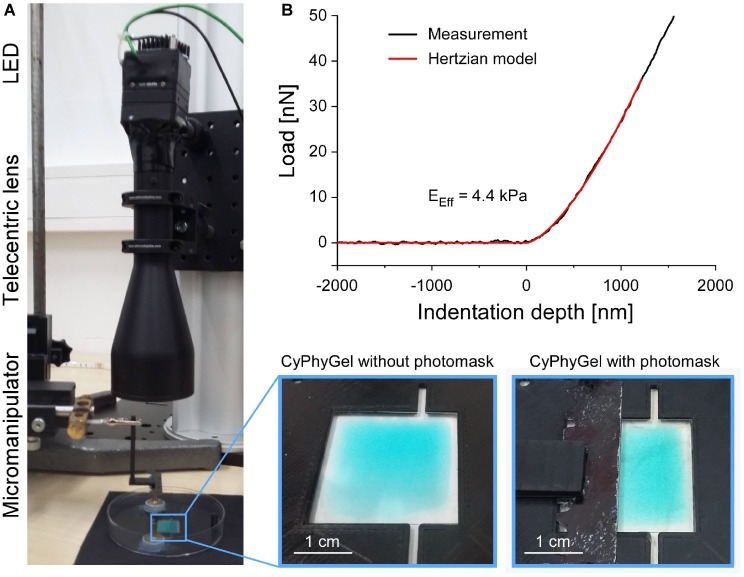
Generation and mechanical testing of heterogeneous CyPhyGels. **(A)** Illumination system for generation of mechanically heterogeneous CyPhyGels. After initial stiffening of a whole CyPhyGel by exposure to 660 nm illumination (final intensity: 1 mW/cm^2^, 5 min), a telecentric lens is positioned in front of a second LED, which is used for 740 nm illumination (final intensity: 1 mW/cm^2^, 5 min) of the partially covered CyPhyGel. **(B)** Representative load-indentation curve resulting from nanoindentation (black) and the fitted Hertzian model for contact mechanics (red) used to calculate the Effective Young’s modulus, *E*_Eff_.

To obtain heterogeneous CyPhyGels, characterized by containing one stiff and one soft half, we implemented a sequential illumination protocol (Figure 1A). After initial stiffening of the whole CyPhyGel by illumination with 660 nm, a photomask (Figure 1A, bottom right) was carefully positioned over half of the gel by means of a micromanipulator. Using a telecentric lens, the exposed part of the CyPhyGel was then illuminated at 740 nm to tune it to its softest state (Figure 1A).

Broad-bandwidth “white” light (e.g. room lighting or microscope light) resulted in stiffening of CyPhyGels in less than 10 min (Supplementary Figure S2). Therefore, CyPhyGels tuned to the desired stiffness were kept in dark conditions or handled under green light. For nanoindentation, the microscope light was filtered with a 490 ± 5 nm band pass filter (D490/10X, Semrock, USA), which prevented gross changes in CyPhyGel stiffness over a period of up to 1 h (Supplementary Figure S2).

### Culture of Primary Human Atrial Fibroblasts

Primary human fibroblasts were obtained from right atrial appendage tissue of patients in sinus rhythm undergoing open heart surgery at the University Heart Center Freiburg-Bad Krozingen. Patients gave informed consent on the use of the tissue (with approval by the Ethics Commission of the University of Freiburg, reference: 393/16: 214/18). Upon excision by the surgeon, the tissue was placed in room temperature cardioplegic solution (containing, in mM 120 NaCl, 25 KCl, 10 HEPES, 10 glucose, 1 MgCl_2_; pH 7.4, 300 mOsm) and immediately transported to the laboratory. After removing the epicardial layer, the tissue was cut into pieces of 1–2 mm^3^. Tissue fragments were placed in culture plates (3.5 cm diameter, 3–4 pieces per well) with abraded surfaces, containing 2 mL Dulbecco’s Modified Eagle Medium supplemented with 10% fetal bovine serum and 1% penicillin/streptomycin (all Sigma-Aldrich, Germany). Medium was refreshed every 3–4 days. Due to their proliferative and migratory potential, fibroblasts are the major cell type growing out of the tissue chunk (Poulet et al., 2016). Immunocytochemical analysis could not detect the presence of endothelial (tested by CD31) or smooth muscle cells (tested by desmin) in the outgrowth cultures (data not shown). The original outgrowing fibroblasts were referred to as “Passage 0.” Once 80–90% confluency was reached, cells were detached with Trypsin-EDTA (Sigma-Aldrich, Germany) and re-cultured (Passage 1) at a density of 6,500 cells/cm^2^. This procedure was repeated up to six times (generating Passages 2–6).

For experiments shown in this manuscript, fibroblasts from three patients at Passages 4 and 5 were seeded at low density (2,000 cells/cm^2^) on previously tuned CyPhyGels. Cell culture work was conducted using green light to limit changes in CyPhyGel stiffness from environmental light exposure. After seeding, culture dishes were covered by aluminum foil during the whole culture period (4 days, no medium change).

### Nanoindentation of CyPhyGels and Fibroblasts

The Effective Young’s modulus, *E*_Eff_, was assessed using the Chiaro nanoindenter system (Optics11, Amsterdam, Netherlands). A spherical tip, attached to a calibrated cantilever, is used to indent the sample while a laser beam is shone onto the reflective cantilever surface. Reflected laser light is analyzed interferometrically to measure phase shifts, which correlate with cantilever bending. From this, the force required for sample indentation is calculated. *E*_Eff_ was derived using the Hertzian model for contact mechanics (Hertz, 1881; Chen, 2014) under the assumption of a Poisson’s ratio of 0.5 for incompressible materials, which is commonly used for mechanical testing of cells and tissue (Figure 1B; Guz et al., 2014). Throughout this manuscript, *E*_Eff_ is referred to as stiffness. Sample indentations of 2–4 μm were performed at a displacement speed of 5 μm/s. Data analysis used the Optics11 DataViewer (V2.0.27).

For CyPhyGel characterization, cantilevers with spring constants of 0.45–0.5 N/m and tips of 20–23 μm radii were used. For cell assessment, cantilevers with spring constants of 0.01–0.02 N/m and tips with 3–3.5 μm radii were used. The stiffness of single cells was determined by performing surface-normal indentations at two to three different positions that were not overlapping with the nucleus. For every indentation, the Hertzian model was used to fit the force-displacement curve from initial cell surface contact up to 1 μm indentation, to avoid mechanical interference from the underlying growth substrate. Cell stiffness was assessed 4 days after seeding on CyPhyGels.

### Cell Fixation, Staining, Confocal Imaging, and Image Analysis

For confocal imaging, fibroblasts were cultured on CyPhyGels of different stiffness for 4 days, fixed using 4% paraformaldehyde (Roth, Germany) in PBS for 15 min. Glycoproteins at the cell membrane were stained with AlexaFluor555-conjugated wheat germ agglutinin (WGA, 1 μg/mL, Invitrogen, Germany) in 3% bovine serum albumin in PBS for 1 h, followed by three 10-min washing steps in PBS. Thereafter, cells were permeabilized using 0.5% Triton-X100 (Sigma-Aldrich, Germany) in PBS for 15 min and blocked using 3% bovine serum albumin in PBS for 1 h. Primary antibody against alpha smooth muscle actin (αSMA; ab7817, Abcam, Germany) was applied 1:50 in 1% bovine serum albumin in PBS overnight. After three washing steps in PBS, AlexaFluor488-coupled secondary anti-mouse was applied 1:500 in PBS for 1 h. Nuclear counterstaining was performed using Hoechst-33342 (20 μM, ThermoFisher, Germany) for 2–3 min in PBS. All staining was performed at room temperature.

Imaging was performed on a Leica TCS SP8 X confocal microscope using a 20× multi-immersion objective (HC PL APO 20x/0.75 IMM) with water as immersion medium. For imaging, the CyPhyGels were mounted upside-down in a PBS-filled microscopy dish (µ-Dish 35 mm, ibidi, Germany). Cell area was determined using ImageJ after manually tracing cell borders based on WGA signal.

### Statistics

If not stated otherwise, data are presented as single data points and mean ± SEM. Data points were considered outliers when they differed by more than three standard deviations from the mean. After removing outliers, statistical significance of two groups with more than 20 data points was determined by Student’s *t*-test for unpaired data with equal variances. Two groups with less than 20 data points were compared using the non-parametric Mann-Whitney test. More than two groups were analyzed by Two-way ANOVA followed by *post-hoc* comparison of the mean values using the Tukey test. All statistical analyses were performed in OriginPro 2019.

## Results

### Tuning Mechanical Properties of CyPhyGels by Light

**Figure 2:**
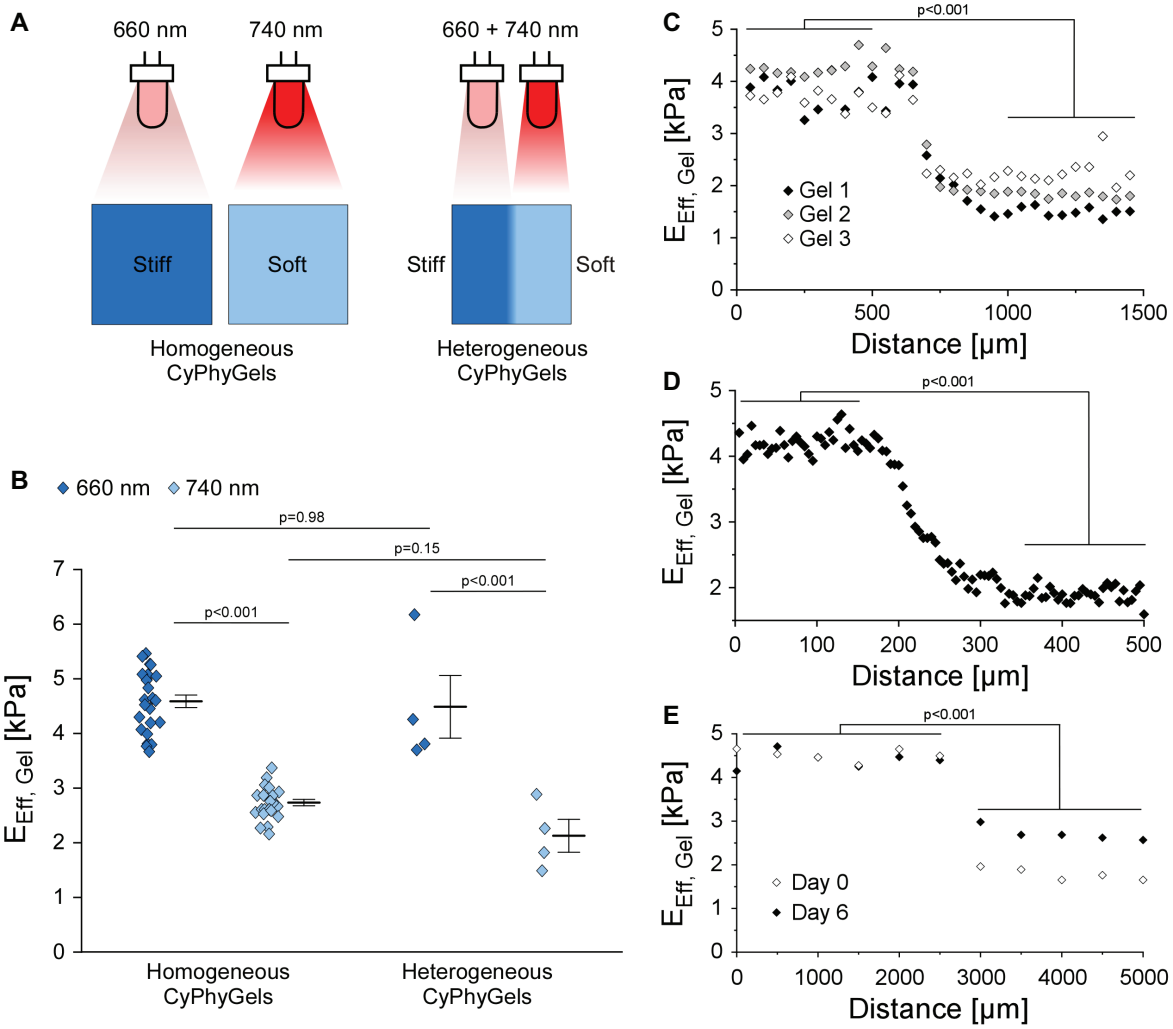
Mechanical properties of CyPhyGels. **(A)** Schematic representation of CyPhyGel configurations used in this experiment. **(B)** *E*_Eff_ of CyPhyGels, exposed to homogeneous illumination with either 660 or 740 nm (*n* = 24) or sequentially illuminated and thus mechanically heterogeneous (*n* = 4), was determined by nanoindentation. **(C)** *E*_Eff_ along an axis perpendicular to the border between stiff and soft halves of three representative heterogeneous CyPhyGels, illustrating the step-change in gel stiffness (50 μm distance between consecutive measurement points, *n* = 3). **(D)** Higher resolution assessment of the transition area in Gel 3 from panel C (5 μm distance between measurement points). **(E)** *E*_Eff_ of Gel 3 from panel C immediately after illumination (Day 0) and 6 days later, corresponding to the duration of an experiment.

CyPhyGels, uniformly exposed to illumination at 660 nm had an *E*_Eff_ of 4.59 ± 0.11 kPa, while after illumination with 740 nm, *E*_Eff_ was 2.72 ± 0.06 kPa (Figures 2A,B). Similar maximal and minimal *E*_Eff_ were found for the two differentially tuned halves of heterogeneous CyPhyGels. For spatial characterization of the stiffness gradient, heterogeneous CyPhyGels were mapped, in 50 μm steps, along a line perpendicular to the boundary between their stiff and soft halves. *E*_Eff_ changed from 4.48 ± 0.58 to 2.12 ± 0.30 kPa (Figure 2C). Using a higher resolution step size (5 μm) near the boundary, we found that the transition between stiff and soft areas occurs within a 100–150 μm wide band (Figure 2D). The width of the border zone was constant over at least 1 mm (Supplementary Figure S3).

To assess whether heterogeneity in CyPhyGel stiffness was sustained over time, stiffness was measured 6 days after sequential illumination, which corresponds to the duration of an experiment. The two areas with different stiffness were still clearly distinguishable (Figure 2E). The slight increase in stiffness in the soft area over 6 days is due to exposure to external light during CyPhyGel handling and cell culture procedures (Supplementary Figure S2).

### Cell Area Analysis

**Figure 3:**
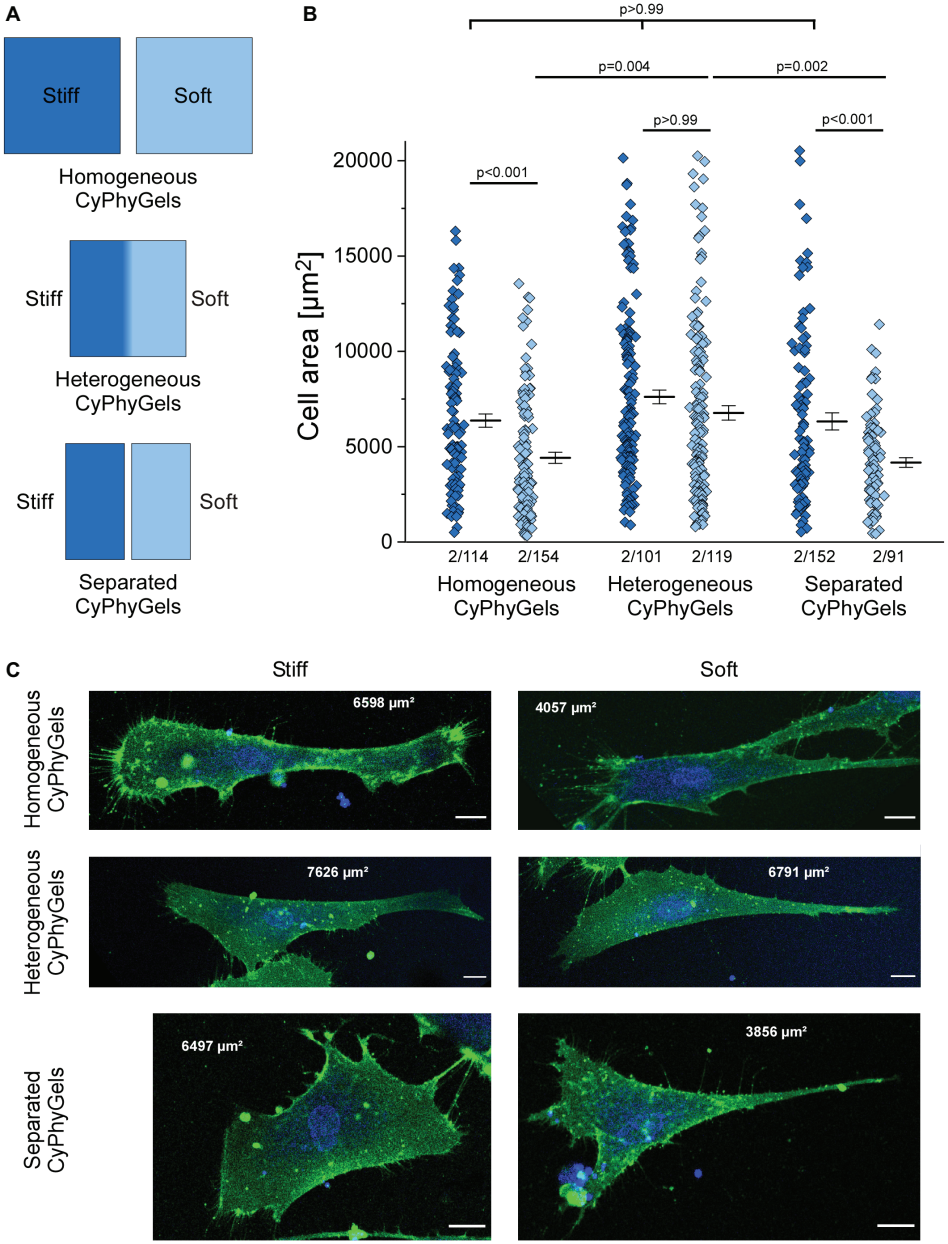
Area of human primary atrial fibroblasts after 4 days of culture on CyPhyGels in different configurations. **(A)** Schematic presentation of CyPhyGels used for the experiments in Figures 3, 4. **(B)** Fibroblasts grown on homogeneously soft CyPhyGels spread less than those grown on homogeneously stiff CyPhyGels. The same applies to separated CyPhyGels, while no difference was found among fibroblasts grown on heterogeneous CyPhyGels (Number of patients/number of cells). **(C)** Representative images of fibroblasts on different CyPhyGels. Green = cell membrane. Blue = nuclear counterstain. Scale bars = 20 μm (Number of patients/number of cells).

One adaptation of fibroblasts to matrix stiffness is the extent of cell spreading, quantified by cell area (Herum et al., 2017). We cultured human primary atrial fibroblasts on CyPhyGels that were tuned to homogeneously stiff, homogeneously soft, or heterogeneous states. While fibroblasts on the stiff CyPhyGels had an average cell area of 6,351 ± 349 μm^2^, the area of fibroblasts on the soft CyPhyGels was 4,398 ± 293 μm^2^ (Figure 3B).

There was no significant difference in cell area occupied by fibroblasts grown on the stiff *versus* the soft half of heterogeneous CyPhyGels (6,563 ± 494 μm^2^ on the stiff half; 6,451 ± 440 μm^2^ on the soft half, Figure 3B). Further, we cultured fibroblasts on CyPhyGels of the same stiffness and area as the two halves of heterogeneous CyPhyGels. These were illuminated homogeneously and one stiff and one soft CyPhyGel were placed next to each other with a separation of 1 mm (Figure 3A, hereafter referred to as separated CyPhyGels). In this setting, the area of cells on the stiff CyPhyGel was higher than those on a soft CyPhyGel (6,306 ± 446 μm^2^ compared to 4,149 ± 251 μm^2^, Figure 3B). Representative images of plasma membrane-stained fibroblasts in all conditions are shown in Figure 3C.

### Cell Stiffness Analysis

**Figure 4:**
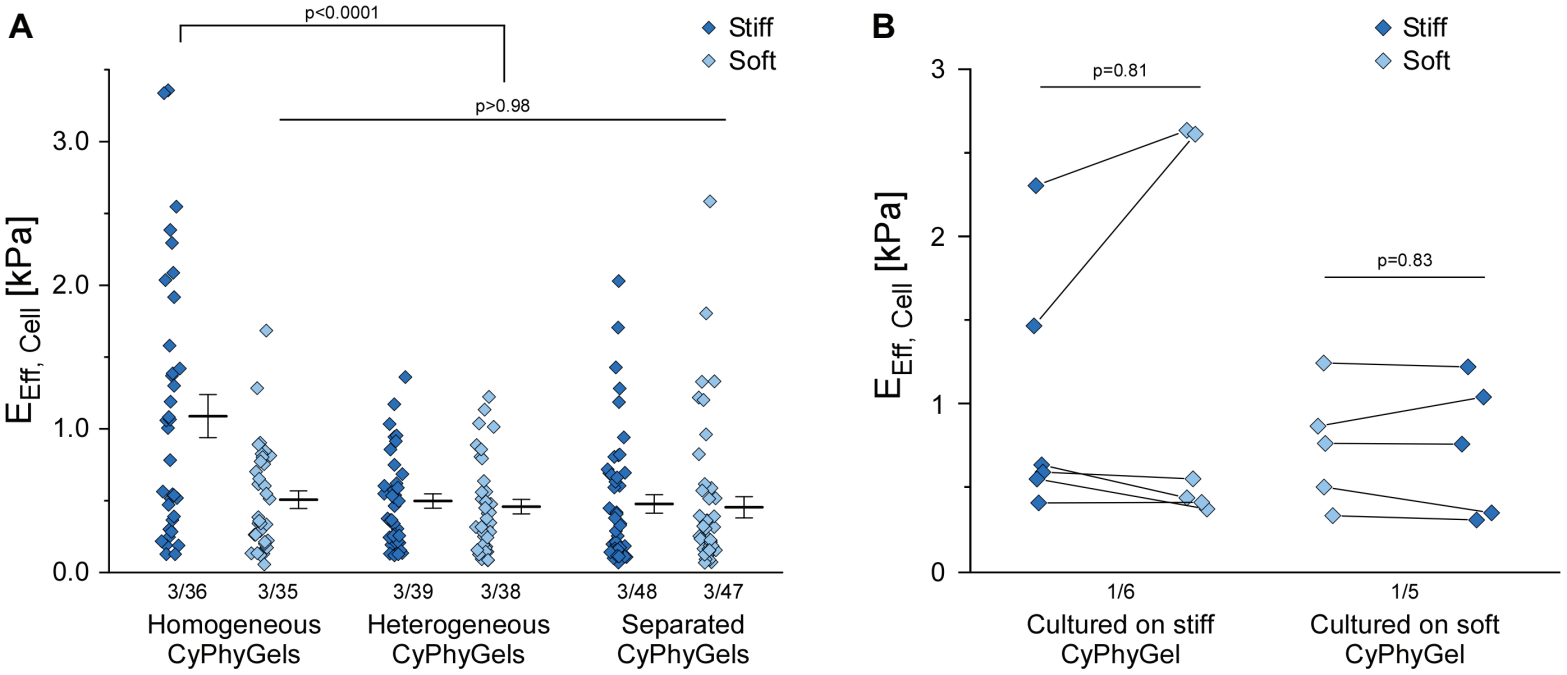
Stiffness of human primary atrial fibroblasts grown on CyPhyGels. **(A)** Fibroblasts grown on homogeneously stiff CyPhyGels are stiffer than those grown on soft CyPhyGels. When cultured on heterogeneous or separated CyPhyGels, fibroblast stiffness is not different from that of fibroblasts on homogeneously soft CyPhyGels. **(B)** *E*_Eff_ of 11 cells was determined before and after an acute change of CyPhyGel stiffness. No systematic difference in recorded cell stiffness was found (Number of patients/number of cells).

Human primary atrial fibroblasts, cultured on stiff CyPhyGels, were stiffer (1.09 ± 0.15 kPa) than those grown on soft CyPhyGels (0.50 ± 0.06 kPa, Figure 4A). Fibroblasts, cultured on heterogeneous CyPhyGels, showed no significant difference in cell stiffness between stiff (0.49 ± 0.05 kPa) and soft areas (0.46 ± 0.05 kPa, Figure 4A). When cultured on separated CyPhyGels, fibroblast stiffness was not different from that on heterogeneous CyPhyGels with 0.47 ± 0.06 kPa on the stiff and 0.45 ± 0.07 kPa on the soft CyPhyGel (Figure 4A). The higher cell stiffness on homogeneously stiff CyPhyGels was not a result of higher αSMA expression levels (Supplementary Figure S4).

To assess any possible contribution of the mechanical properties of the CyPhyGel on cell stiffness measurements, we acutely reversed CyPhyGel stiffness. Switching CyPhyGels from stiff to soft state and *vice versa* takes less than 1 min (Supplementary Figure S5). Cells, grown on a homogeneously stiff CyPhyGel, were assessed before and after switching the CyPhyGel to its soft state, and *vice versa.* Cell stiffness before and after switching did not differ significantly, neither for cells initially cultured on stiff (0.99 ± 0.30 kPa before *versus* 1.17 ± 0.46 kPa after acute stiffness reversal) or soft CyPhyGels (0.74 ± 0.16 kPa before *versus* 0.73 ± 0.18 kPa after stiffness reversal, Figure 4B).

## Discussion

Cardiac fibrosis is a multifactorial process accompanying most cardiac malfunctions, and it increases with age. It is characterized by extensive accumulation of ECM proteins, as well as differentiation of fibroblasts into myofibroblasts, both adding to changes in tissue mechanics. Many studies in the past have addressed the questions of how cells sense and respond to the mechanics of their environment. These studies are usually based on model systems in which cells are cultured on substrates with homogeneous mechanical properties.

In this study, we used a novel hydrogel system, which allows one to dynamically change substrate stiffness by illumination with either 660 nm or 740 nm light. We show that CyPhyGel stiffness (*E*_Eff_) can be changed between ~4.6 and ~2.7 kPa, in line with previous results by Hörner et al. (2019b). We confirm the utility of CyPhyGels in experimental conditions involving the cell culture of human primary atrial fibroblasts.

The light-induced stiffness difference is biologically relevant, as we found human primary atrial fibroblasts adapt to the higher substrate stiffness with increased cell area and stiffness. For healthy cardiac tissue, elastic moduli from 5 to 20 kPa have been described, while under pathologic conditions, the elastic modulus of the tissue changes dynamically. During the inflammatory phase following myocardial infarction, tissue stiffness in the infarct area is decreased due to collagen degradation and death of cardiomyocytes. This is followed by massive collagen deposition over weeks leading to an increase in tissue stiffness to more than 55 kPa, depending on species, form of fibrosis, and duration of tissue remodeling (Holmes et al., 2005; Berry et al., 2006; Gluck et al., 2017; Farré et al., 2018). In contrast, the elastic modulus of *in vitro* mouse fibroblast spheroids was determined to be in the range of 0.5–3.5 kPa (Jorgenson et al., 2017). Thus, there are significant differences between the relevant stiffness ranges for *in vivo* and *in vitro* systems, and the here presented CyPhyGels have a relevant dynamic range.

In this study, fibroblasts were also cultured on CyPhyGels with heterogeneous stiffness. The stiffnesses of the two sides of heterogeneous CyPhyGels were the same as for homogeneously illuminated CyPhyGels. The boundary between stiff and soft halves was well defined, confined to ~150 μm, i.e., on a par with the size of one or two cultured fibroblasts.

While fibroblasts on homogeneous CyPhyGels adapt to their culture substrate with higher cell area and stiffness than those on the stiff CyPhyGel, such adaptation was not present on heterogeneous CyPhyGels. In terms of cell area, the fibroblasts on heterogeneous CyPhyGels are as large as fibroblasts on homogeneously stiff CyPhyGels, while cell stiffness resembles that of fibroblasts on homogeneously soft CyPhyGels. To figure out whether this effect was mediated by direct cell–cell contact or *via* secreted mediators, we performed the same experiments with a physical separation of 1 mm between the stiff and the soft CyPhyGel. In this setting, fibroblasts were not able to directly contact each other, while secretory mediators were transmitted *via* the culture medium. Surprisingly, we found an adaptation of cell area to matrix stiffness on separated CyPhyGels, while cell stiffness was still the same as on homogeneously soft CyPhyGels. Our results suggest that the adaptation of cell spreading and cell stiffness in response to matrix stiffness are regulated by distinct mechanisms, where cell spreading can be modulated by cell-cell contact, while cell stiffness can be modulated by paracrine mediators. Several secreted factors, including transforming growth factor β, angiotensin and interleukins, have been described to play a role in cardiac fibrosis by upregulating collagen production or driving myofibroblast differentiation (Wang et al., 2016; Zhang et al., 2016; Khalil et al., 2017; Schafer et al., 2017; Kuragano et al., 2018). On the other hand, anti-fibrotic effects have been described for conditioned medium from bone marrow-derived stem cells and cardiac progenitor cells and may involve secreted growth factors and/or microRNAs (Kishore et al., 2013; Fang et al., 2016). Further experiments will be necessary to identify the mechanisms responsible for the effects observed in this study.

The higher cell area and stiffness observed on stiffer substrates can be interpreted as indicative of differentiation toward a myofibroblastic phenotype. However, we did not find a higher proportion of fibroblasts expressing the myofibroblast indicator αSMA on stiff CyPhyGels. This might be explained by a potential pre-activation of fibroblasts during their culture time on plastic and the low range of stiffnesses that is covered by CyPhyGels. Current literature on mechanical control of fibroblast phenoconversion usually utilizes larger stiffness differences (Sunyer et al., 2012; Herum et al., 2017), which might trigger increased αSMA expression more efficiently. Also, there are some discrepancies regarding the regulation of αSMA expression response to matrix stiffness. While usually αSMA is thought to be upregulated with higher matrix stiffness, the opposite was also shown for human valve interstitial fibroblasts (Mabry et al., 2015). This, in line with our results, indicates that the regulation of fibrotic events in response to matrix stiffness is highly complex and involves many factors. Distinct phenotypic properties seem to be regulated by different stimuli. Future work will assess, for example, how culturing on plastic may affect mechanosensing in fibroblasts. All in all, more studies will be required to integrate the results described here with previous knowledge on fibrotic mechanisms gathered from mechanically invariable substrates.

CyPhyGels, by enabling spatial and temporal alterations in the stiffness of the growth substrate, will aid the design and implementation of improved experimental models for studying the basic mechanisms underlying fibrosis. We show that fibroblasts can be grown on CyPhyGels of different stiffness, and that they sense and respond to the stiffness changes that can be imparted by CyPhyGels. Further studies will assess the speed at which cells respond to stiffness changes, explore whether fibrosis can be reversed by altering substrate stiffness, and whether mechanical memory plays a role in fibrosis.

## Conclusion

We present a novel *in vitro* model suitable for the study of dynamic biological responses to local and temporal changes in matrix stiffness. This model will be used to shed light on mechanisms of communication between cells in differing mechanical microenvironments, for example to mimic fibrotic or scarred myocardium. Our results indicate that cardiac fibroblast adaptations to mechanical properties of the growth matrix may be able to be tuned *via* paracrine factors as well as direct cell-cell contact.

## Data Availability Statement

The datasets generated for this study are available on request to the corresponding author.

## Ethics Statement

The studies involving human participants were reviewed and approved by Ethics Commission of the University of Freiburg, Freiburg, Germany (reference: 393/16: 214/18). The patients/participants provided their written informed consent to participate in this study.

## Author Contributions

PK, RP, CZ-J, UR, and RE contributed to the conception and design of the study. MH and WW developed the CyPhyGels and supported its experimental application. FB and BR provided access to surgical tissue samples. RE performed and analyzed the experiments. RE, CZ-J, and RP drafted the manuscript. All authors contributed to manuscript revision, read and approved the submitted version.

### Conflict of Interest

The authors declare that the research was conducted in the absence of any commercial or financial relationships that could be construed as a potential conflict of interest.

## Supplementary Material

SUPPLEMENTARY FIGURE S1 After preparation, CyPhyGels were stored in PBS at room temperature in darkness. Their stiffness in response to 660 or 740 nm illumination was determined over 40 days after preparation. After 1 week, CyPhyGel stiffness is reversibly tunable between ~3 and ~5 kPa for at least 40 days (*n* = 3).

SUPPLEMENTARY FIGURE S2 *E*_Eff_ of CyPhyGels exposed to different light regimes. When exposed to green background light only, soft-tuned CyPhyGels stayed soft for at least 1 h. Exposure to white light (e.g. microscope or room light, >10 mW/cm^2^) resulted in complete stiffening of the CyPhyGel within 10 min. Filtering the microscope light using a 490 ± 5 nm band pass filter (final light intensity ~0.05 mW/cm^2^), minimized the stiffening for at least 1 h. All experiments were performed under green background light.

SUPPLEMENTARY FIGURE S3 2D mapping of E_Eff_ of a heterogeneous CyPhyGel in a squared area of 1 mm × 1 mm, including the border zone, measured with a step size of 50 μm. The left part of the mapped area is stiffer than the right part. The border zone is well delineated and about 100–150 μm wide.

SUPPLEMENTARY FIGURE S4 αSMA expression in human primary atrial fibroblasts cultured on different CyPhyGels. **(A)** Fibroblasts were fixed in 4% paraformaldehyde and immunostained for αSMA after 4 days in culture on CyPhyGels in different configurations. Red = αSMA. Blue = nuclear counterstain. Scale bars = 150 μm. **(B)** αSMA-positive cells of two patients were determined as percentage of total cells after applying the same intensity threshold to all images.

SUPPLEMENTARY FIGURE S5 Kinetics of CyPhyGel stiffness switch in response to changed illumination. At 50 s, the illumination was switched from 660 to 740 nm or *vice versa*. As controls, CyPhyGels were constantly illuminated with either of the two respective wavelengths. All illuminations were performed at 1 mW/cm^2^.
