# Supplementary figures and images for: Human Atrial Fibroblast Adaptation to Heterogeneities in Substrate Stiffness
Source: Front Physiol. 2020 Jan 10;10:1526. doi: 10.3389/fphys.2019.01526 (PMC6965062; doi:10.3389/fphys.2019.01526)

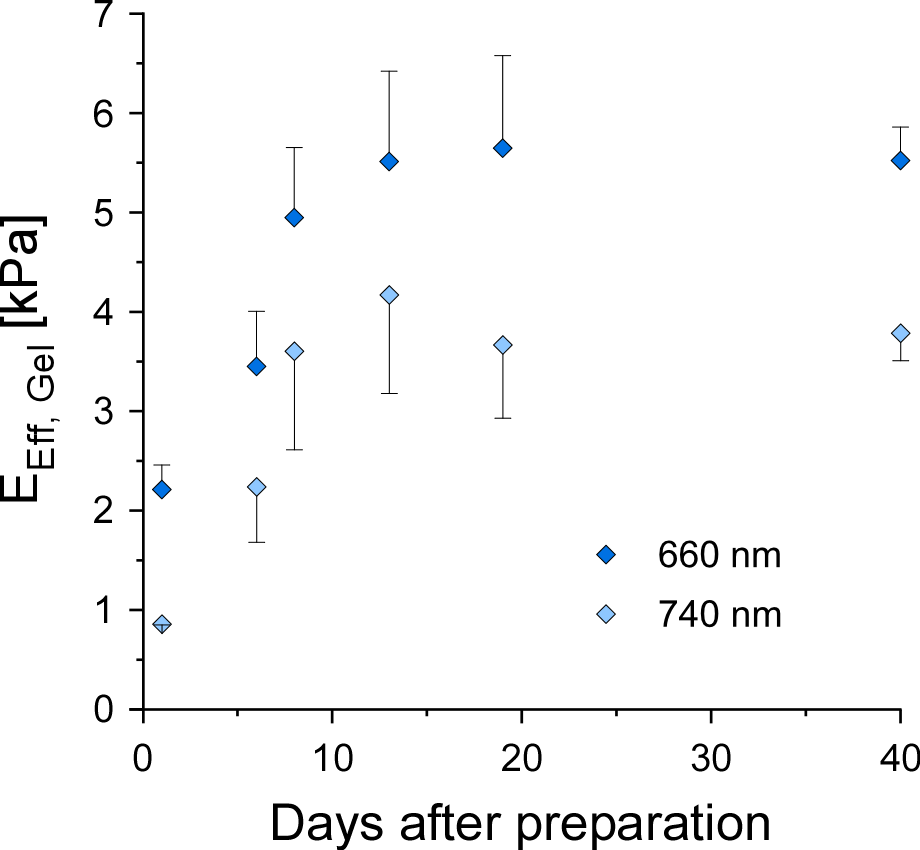

Supplement: SUPPLEMENTARY FIGURE S1 — After preparation, CyPhyGels were stored in PBS at room temperature in darkness. Their stiffness in response to 660 or 740 nm illumination was determined over 40 days after preparation. After 1 week, CyPhyGel stiffness is reversibly tunable between ~3 and ~5 kPa for at least 40 days (n = 3). [file Presentation_1.zip › Presentation_1/Sup/Figure S1.TIF]

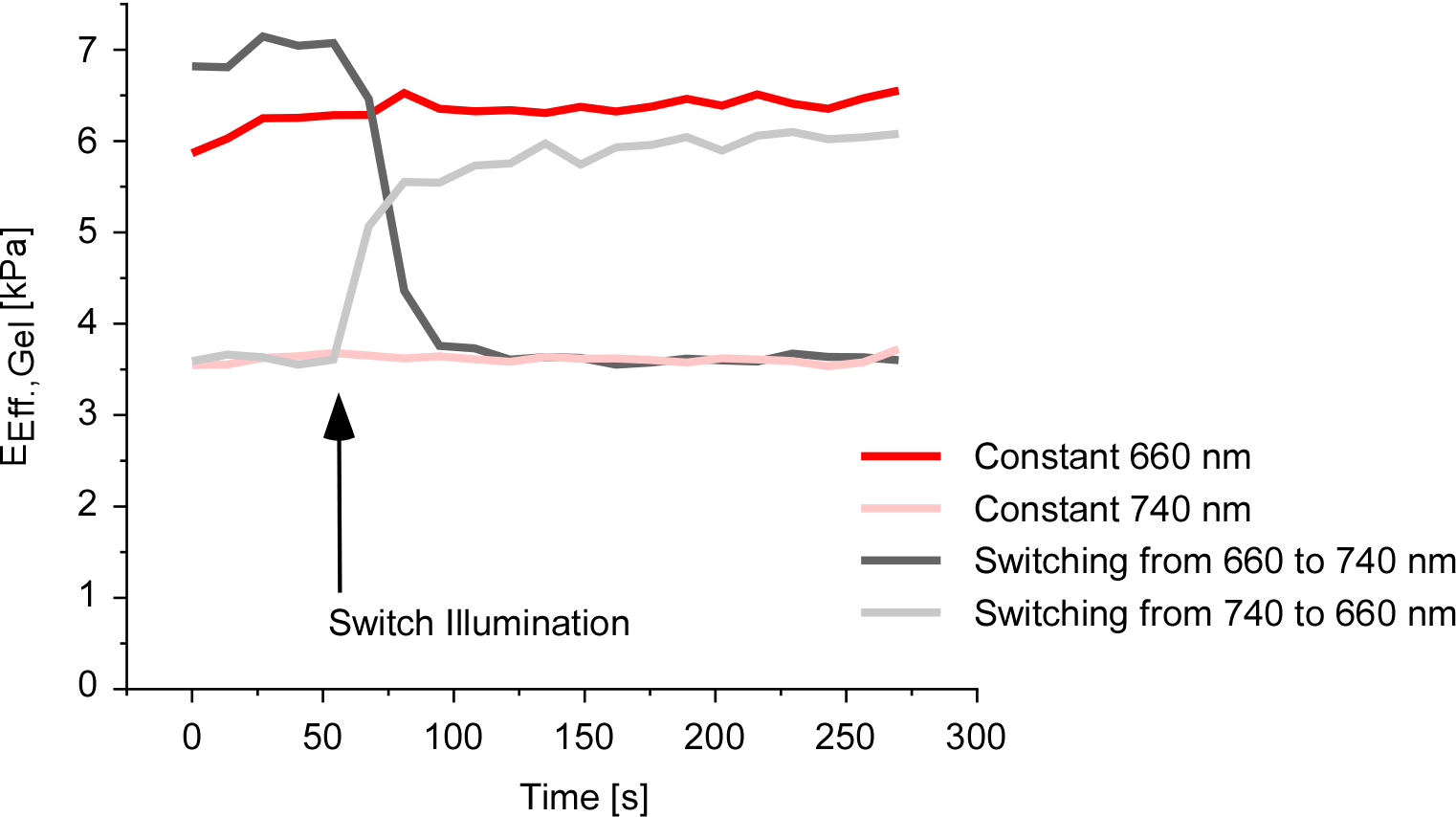

Supplement: SUPPLEMENTARY FIGURE S1 — After preparation, CyPhyGels were stored in PBS at room temperature in darkness. Their stiffness in response to 660 or 740 nm illumination was determined over 40 days after preparation. After 1 week, CyPhyGel stiffness is reversibly tunable between ~3 and ~5 kPa for at least 40 days (n = 3). [file Presentation_1.zip › Presentation_1/Sup/Figure S5.TIF]

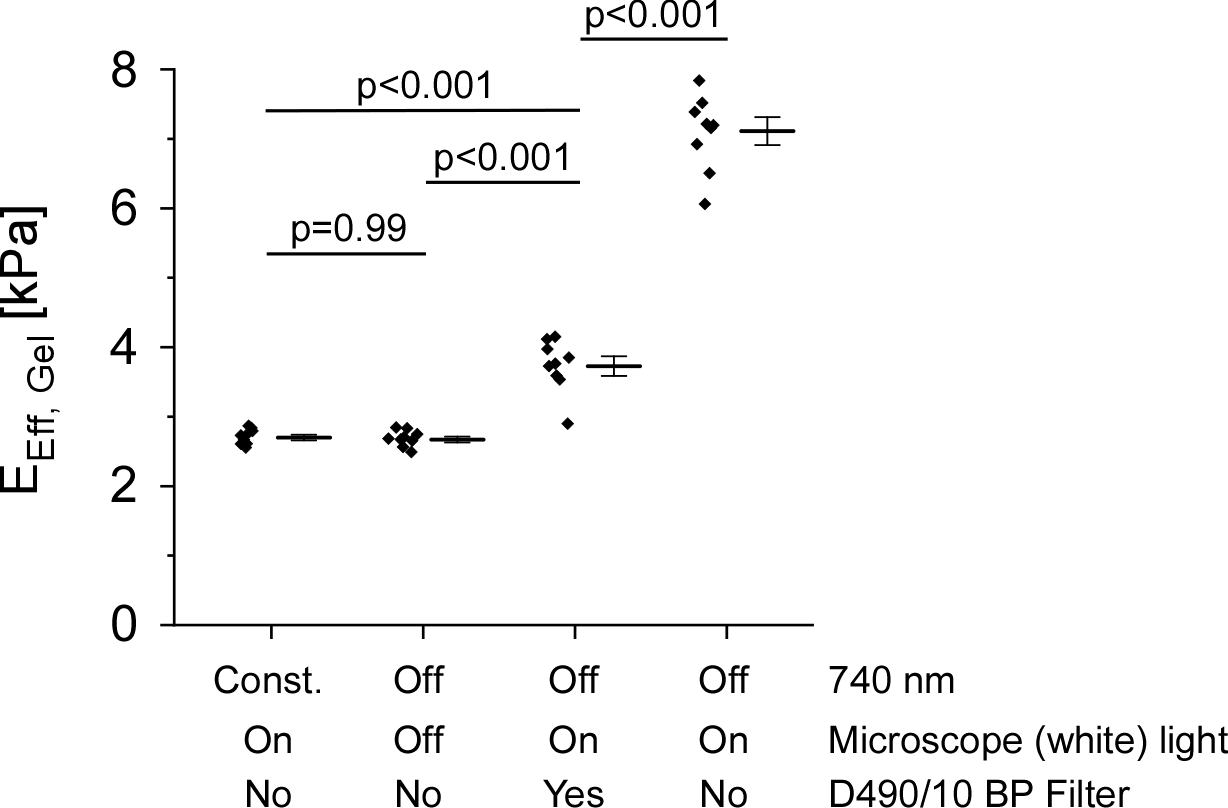

Supplement: SUPPLEMENTARY FIGURE S1 — After preparation, CyPhyGels were stored in PBS at room temperature in darkness. Their stiffness in response to 660 or 740 nm illumination was determined over 40 days after preparation. After 1 week, CyPhyGel stiffness is reversibly tunable between ~3 and ~5 kPa for at least 40 days (n = 3). [file Presentation_1.zip › Presentation_1/Sup/Figures S2.TIF]

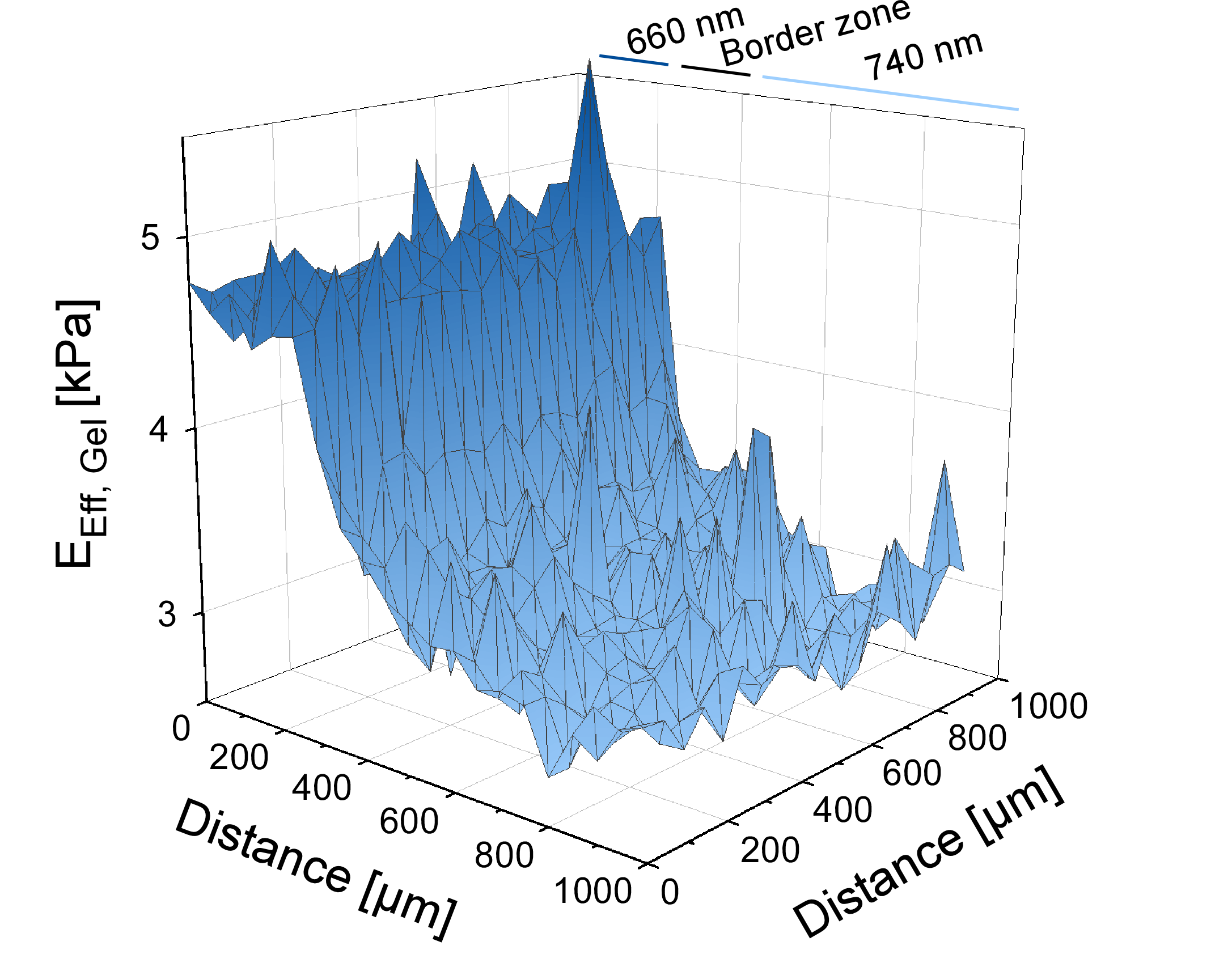

Supplement: SUPPLEMENTARY FIGURE S1 — After preparation, CyPhyGels were stored in PBS at room temperature in darkness. Their stiffness in response to 660 or 740 nm illumination was determined over 40 days after preparation. After 1 week, CyPhyGel stiffness is reversibly tunable between ~3 and ~5 kPa for at least 40 days (n = 3). [file Presentation_1.zip › Presentation_1/Sup/FigureS3.tif]

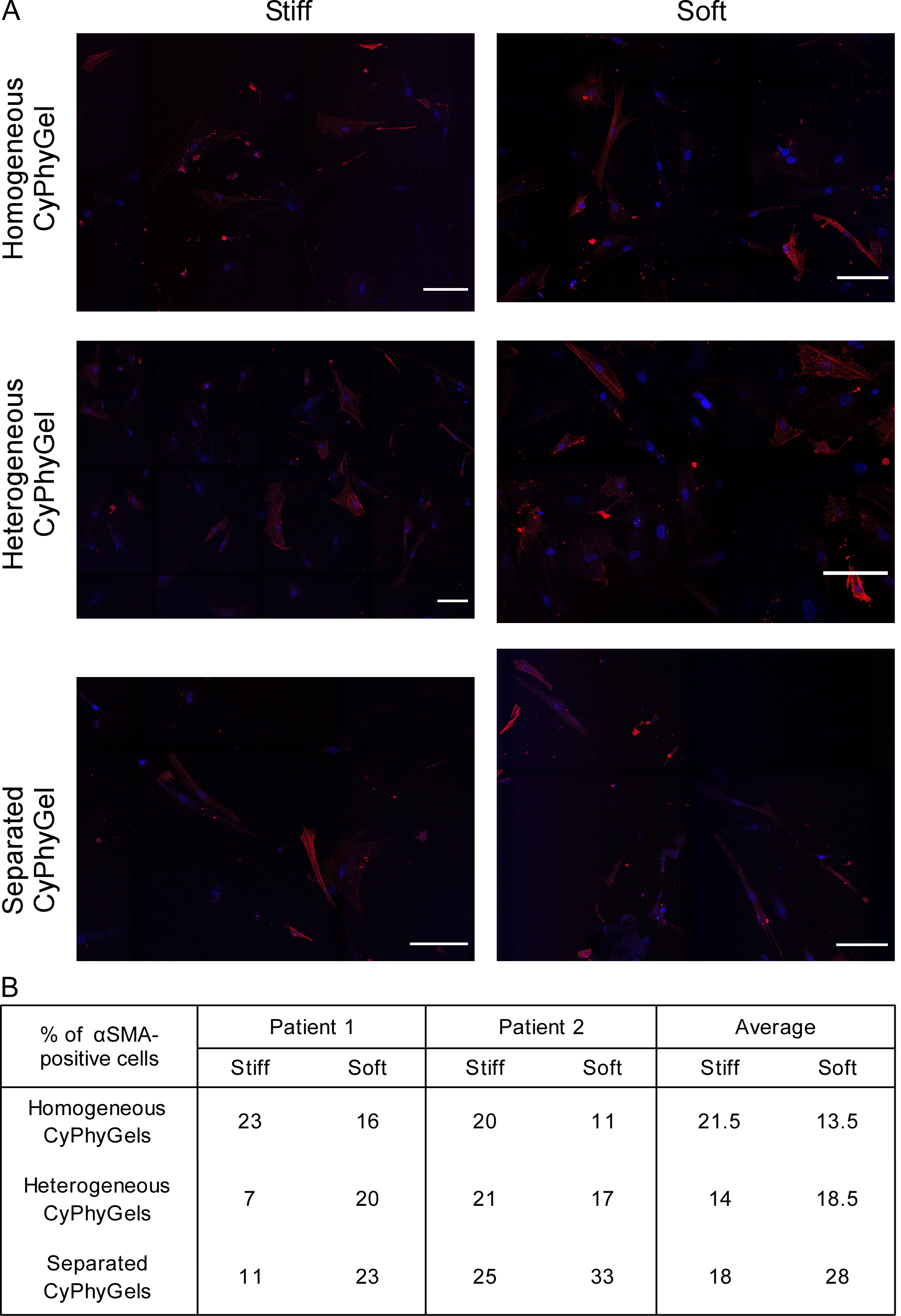

Supplement: SUPPLEMENTARY FIGURE S1 — After preparation, CyPhyGels were stored in PBS at room temperature in darkness. Their stiffness in response to 660 or 740 nm illumination was determined over 40 days after preparation. After 1 week, CyPhyGel stiffness is reversibly tunable between ~3 and ~5 kPa for at least 40 days (n = 3). [file Presentation_1.zip › Presentation_1/Sup/FigureS4.tif]
